# The Impact of Drug Delivery Systems on Pharmacokinetics and Drug-Drug Interactions in Neuropsychiatric Treatment

**DOI:** 10.7759/cureus.85563

**Published:** 2025-06-08

**Authors:** Mohammad Nammas

**Affiliations:** 1 School of Pharmacy, University of Pittsburgh, Pittsburgh, USA

**Keywords:** drug delivery, drug-drug interaction, neuropsychiatric, personalized medicine, pharmacokinetics

## Abstract

Neuropsychiatric drugs are essential for the management of mental health disorders, yet their therapeutic effectiveness is often compromised by complex pharmacokinetics (PK), significant drug-drug interactions (DDIs), limited blood-brain barrier (BBB) penetration, and poor patient adherence. Advanced drug delivery systems (DDSs) offer innovative strategies to overcome these challenges by improving drug solubility, targeting, release kinetics, and systemic exposure profiles. This review critically examines the impact of DDS on the PK and DDI profiles of psychotropic medications, highlighting key technologies such as nanoparticles, transdermal systems, prodrugs, depot formulations, implantables, and smart delivery platforms. By enhancing drug bioavailability and CNS targeting while minimizing systemic toxicity and DDI risks, these systems enable more consistent therapeutic outcomes across diverse neuropsychiatric conditions. This review also explores the integration of DDS with personalized medicine approaches - pharmacogenomics, biomarker-driven delivery, and artificial intelligence - to facilitate tailored treatment strategies. Furthermore, it addresses practical challenges including regulatory hurdles, cost and accessibility, long-term safety data, and patient education. Through interdisciplinary innovation and strategic implementation, DDSs are poised to revolutionize neuropsychiatric pharmacotherapy by offering safer, more effective, and patient-centric solutions.

## Introduction and background

Neuropsychiatric disorders constitute a major global health burden, with complex etiologies and heterogeneous clinical presentations that complicate diagnosis and management. As of 2023, the World Health Organization (WHO) estimates that nearly one billion individuals, approximately one in eight globally, are affected by a mental health condition, a figure exacerbated by the COVID-19 pandemic, sociopolitical instability, and widening healthcare disparities [1]. Conditions such as major depressive disorder, anxiety disorders, bipolar disorder, and schizophrenia account for over 14% of age-standardized years lived with disability (YLDs), reflecting both their chronicity and profound functional impact [2]. Beyond individual suffering, these disorders impose substantial societal costs in terms of healthcare expenditure, lost productivity, and reduced quality of life [3,4].

Despite the availability of various pharmacological and psychotherapeutic modalities, treatment outcomes for many patients remain suboptimal. High rates of partial response, treatment resistance, and poor adherence to long-term therapy persist across diagnostic categories [5]. In low- and middle-income countries (LMICs), structural barriers further exacerbate access issues, with over 75% of affected individuals receiving no formal treatment [6]. Even in well-resourced settings, the effectiveness of pharmacotherapy is frequently attenuated by unfavorable pharmacokinetic (PK) properties, limited blood-brain barrier (BBB) permeability, systemic side effects, and polypharmacy-induced drug-drug interactions (DDIs) [7-9]. Additionally, the emergence of precision medicine has highlighted the inadequacy of empiric, population-level dosing regimens, particularly in psychiatry, where genetic variations in drug-metabolizing enzymes like CYP2D6 and CYP2C19 can lead to significant differences in therapeutic response and adverse effects.

Advanced drug delivery systems (DDSs) have emerged as a pivotal innovation to address these limitations. By engineering how and where a drug spreads in the body over time, DDS optimizes drug solubility, release kinetics, and tissue targeting while minimizing systemic toxicity and metabolic degradation [10,11]. Innovations such as nanocarriers, transdermal systems, liposomal encapsulation, prodrug strategies, and implantable devices provide differentiated release profiles and improved pharmacodynamic engagement of neuropsychiatric agents [12-17]. Notably, nanoscale formulations have demonstrated enhanced BBB penetration via receptor-mediated transcytosis and endocytic mechanisms, allowing for targeted central nervous system (CNS) delivery. Similarly, transdermal and subcutaneous systems circumvent hepatic first-pass metabolism and gastrointestinal degradation, enabling improved bioavailability and reduced dosing frequency [18,19]. DDS are particularly beneficial for APIs with narrow therapeutic windows, high first-pass metabolism, or poor aqueous solubility.

**Figure 1:**
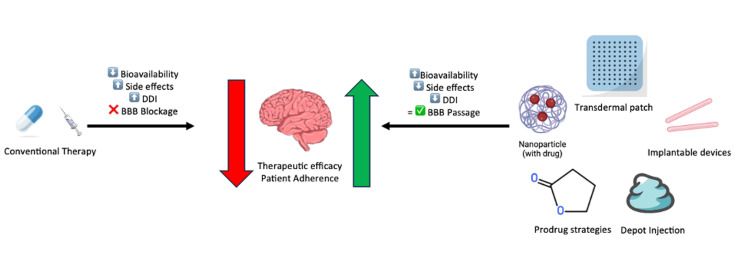
Comparison of conventional therapies and advanced drug delivery systems in terms of bioavailability, side effects, drug-drug interactions (DDIs), and blood-brain barrier (BBB) passage. Conventional therapies are often limited by low bioavailability, high side effects, drug-drug interactions (DDIs), and poor blood-brain barrier (BBB) penetration, resulting in reduced therapeutic efficacy and patient adherence. In contrast, advanced delivery systems - including nanoparticles, prodrug strategies, depot injections, transdermal patches, and implantable devices - enhance BBB passage, improve bioavailability, and reduce side effects. The image is created by the author of this study.

By enhancing therapeutic indices, reducing interindividual variability, and enabling novel routes of administration, these systems can extend the clinical utility of psychotropic compounds previously limited by unfavorable PK or tolerability profiles. Treatment-resistant depression, psychotic disorders with metabolic comorbidities, and neuropsychiatric syndromes with co-occurring substance use represent priority areas for DDS intervention. Figure 1 compares conventional therapies and advanced drug delivery systems in terms of bioavailability, side effects, DDIs, and BBB passage.

Nevertheless, the successful translation of DDS into clinical practice remains constrained by several barriers, including high development costs, regulatory complexity, manufacturing scalability, and the paucity of long-term safety data [20]. Moreover, the integration of pharmacogenomics, digital health technologies, and artificial intelligence (AI) into DDS design is rapidly advancing the field toward precision-guided, adaptive delivery platforms [21-24]. These approaches facilitate modeling of drug-excipient compatibility, simulation of CNS penetration, and real-time adherence monitoring, aligning drug release with individual biological profiles.

This review provides a comprehensive analysis of DDS technologies in the context of neuropsychiatric therapeutics, with particular emphasis on their influence on pharmacokinetics, DDI mitigation, and patient adherence. Key sections address the mechanistic basis of emerging DDS, regulatory and implementation barriers, and opportunities for integration with personalized medicine frameworks. The ultimate objective is to highlight how DDS may overcome critical limitations in current neuropsychiatric pharmacotherapy and catalyze a shift toward more effective, safe, and individualized treatment paradigms [25].

## Review

Methodology

This review was designed to synthesize contemporary advancements in drug delivery systems (DDSs) that enhance pharmacokinetic (PK) profiles and mitigate drug-drug interactions (DDIs) in neuropsychiatric pharmacotherapy. A structured literature search and selection protocol was employed to ensure comprehensive coverage of innovative DDS modalities with translational or clinical relevance.

Literature Search Strategy

A systematic literature search was conducted across the following four major databases: PubMed, Scopus, Web of Science, and Google Scholar, encompassing publications from January 2010 to December 2024. Both MeSH terms and free-text keywords were utilized, including “drug delivery systems,” “neuropsychiatric drugs,” “pharmacokinetics,” “blood-brain barrier,” “drug-drug interactions,” “nanoparticles,” “transdermal systems,” “prodrugs,” “depot injections,” “implantable systems,” and “extended-release.” Boolean operators and database-specific filters were applied to refine results to English-language, peer-reviewed articles involving human studies or relevant preclinical models.

Inclusion and Exclusion Criteria

Studies were included if they focused on neuropsychiatric conditions, such as depression, schizophrenia, bipolar disorder, or attention-deficit/hyperactivity disorder (ADHD); evaluated drug delivery system (DDS) platforms that influence pharmacokinetic (PK) parameters, such as absorption, distribution, metabolism, and excretion, or aimed to reduce drug-drug interactions (DDIs); and comprised preclinical studies with translational value, clinical trials, systematic reviews, or high-impact narrative reviews offering mechanistic or therapeutic insights. Studies were excluded if they did not address central nervous system (CNS) disorders or drug delivery technologies, consisted of editorials, commentaries, or opinion pieces lacking supporting data, or were non-English publications or those with inaccessible full texts.

Selection and Data Extraction

From an initial pool of over 500 publications, duplicates were removed, and titles/abstracts were screened for relevance. Full-text review of shortlisted articles led to the inclusion of approximately 120 studies. Extracted data included the type and mechanism of the drug delivery system (DDS), such as nanocarriers, transdermal patches, or prodrugs; the targeted neuropsychiatric condition; reported clinical or preclinical outcomes, including effects on bioavailability, blood-brain barrier penetration, and mitigation of drug-drug interactions (DDIs); and pharmacokinetic modifications, such as changes in half-life, C-max, or metabolic pathways.

This approach ensured a rigorous and focused analysis aligned with the review’s objective to highlight how DDS innovations are transforming the pharmacological management of mental health disorders through enhanced safety, efficacy, and personalized treatment potential.

Advancements in drug delivery systems

DDSs represent a major leap in overcoming pharmacokinetic challenges by tailoring drug delivery and optimizing therapeutic profiles. Key innovations are discussed further below.

Nanotechnology-Based DDS

Nanotechnology-based DDSs have introduced a transformative framework for addressing the pharmacokinetic limitations of conventional psychotropic agents, particularly those associated with poor solubility, rapid metabolism, and limited central nervous system (CNS) bioavailability. Engineered nanocarriers, including liposomes, polymeric micelles, dendrimers, solid lipid nanoparticles (SLNs), and nanostructured lipid carriers (NLCs), facilitate the encapsulation, protection, and targeted delivery of neuroactive compounds, thereby enhancing therapeutic indices and minimizing off-target effects [26,27].

A principal advantage of nanoscale DDS lies in their capacity to traverse the blood-brain barrier (BBB), a formidable physiological obstacle that limits CNS penetration of most small molecules and nearly all biologics. Nanocarriers exploit active transport mechanisms, such as receptor-mediated transcytosis (e.g., via transferrin or insulin receptors), carrier-mediated transport, or adsorptive-mediated endocytosis, to deliver encapsulated agents to specific CNS regions with high precision [28,29]. Functionalization with surface ligands, including antibodies, peptides, or aptamers, further enhances targeting specificity and reduces non-specific biodistribution.

Liposomes have demonstrated clinical utility due to their bilayer architecture, which accommodates both hydrophilic and lipophilic agents. Liposomal risperidone formulations, for example, enhance CNS delivery while reducing extrapyramidal and metabolic side effects, likely through prolonged systemic circulation and preferential accumulation in brain tissues [30]. Similarly, SLNs and NLCs improve the pharmacokinetic profiles of lipophilic anti-psychotics by enhancing solubility, reducing first-pass metabolism, and enabling controlled release [31].

Polymeric micelles, self-assembled from amphiphilic block copolymers, offer a high-loading platform for hydrophobic drugs with poor aqueous solubility. These structures improve plasma stability and prolong circulation half-life, while enabling stimulus-sensitive release profiles. Several studies have demonstrated the utility of polymeric micelles in delivering selective serotonin reuptake inhibitors (SSRIs) and tricyclic anti-depressants with enhanced brain accumulation and reduced peripheral exposure [32].

Stimuli-responsive nanocarriers represent a major advancement in precision neurotherapeutics. These "smart" systems are engineered to release their cargo in response to pathological microenvironmental cues such as pH gradients, redox potential, enzymatic activity, or temperature shifts. In neuropsychiatric disorders, pH-sensitive nanoparticles have been explored for selective drug release in acidic microenvironments associated with neuroinflammation or oxidative stress [33].

Nanogels - crosslinked, hydrogel-based nanoparticles - enable multidrug loading and controlled release through swelling, degradation, or stimulus-triggered conformational changes. Their high biocompatibility and responsiveness to multiple stimuli (e.g., ionic strength, pH, enzymes) render them particularly suited for CNS applications requiring fine-tuned dosing. Theranostic nanocarriers integrate diagnostic imaging agents (e.g., fluorophores, radionuclides, MRI contrast materials) alongside therapeutic payloads, enabling real-time monitoring of drug distribution, release kinetics, and therapeutic response [34,35].

Despite their promise, nanocarriers face translational barriers including complex manufacturing protocols, scale-up challenges, potential immunogenicity, and incomplete long-term safety profiles. Regulatory classification remains uncertain for multifunctional systems that straddle drug, device, and diagnostic categories. Nonetheless, ongoing clinical trials and platform standardization efforts are gradually mitigating these constraints.

In sum, nanotechnology-based DDS offers a versatile and potent solution to the multifactorial delivery challenges inherent in neuropsychiatric pharmacotherapy. By enabling BBB penetration, sustained and site-specific release, and biomarker-responsiveness, these systems hold significant potential to improve therapeutic efficacy, safety, and precision in the management of psychiatric disorders.

Transdermal Drug Delivery

Transdermal drug delivery systems (TDDS) offer a non-invasive and physiologically advantageous route for systemic drug administration, circumventing gastrointestinal degradation and hepatic first-pass metabolism while enabling sustained and controlled drug release [36]. For psychotropic agents, TDDSs provide a means to maintain steady-state plasma concentrations, reduce dosing frequency, and enhance patient adherence, particularly in chronic psychiatric conditions characterized by fluctuating symptomatology and poor medication compliance.

The pharmacokinetic rationale for TDDS is grounded in their ability to deliver drugs across the stratum corneum, into the viable epidermis and dermis, and subsequently into the systemic circulation. Drug permeation is governed by physicochemical parameters, including molecular weight (ideally <500 Da), lipophilicity (log P between 1 and 3), and low dose requirements. These criteria align with several psychotropic agents, enabling successful transdermal formulations in clinical use.

A well-established example is the selegiline transdermal system (EMSAM), used in the treatment of major depressive disorder (MDD). This monoamine oxidase B inhibitor bypasses the gastrointestinal tract, allowing for higher systemic and CNS drug levels while avoiding tyramine-related hypertensive crises associated with oral administration. The steady plasma concentration achieved through the transdermal route has been associated with enhanced anti-depressant efficacy and improved tolerability profiles [37].

Beyond passive systems, advanced TDDS platforms incorporate technologies such as microneedles, iontophoresis, and electroporation to overcome the skin’s barrier function and expand the range of deliverable compounds. Microneedle arrays - composed of dissolvable or coated biodegradable polymers - create microchannels in the skin to facilitate the delivery of larger molecules or poorly permeable drugs. These systems are currently under investigation for delivery of anti-psychotics and mood stabilizers with favorable outcomes in early-phase clinical trials [38,39].

Hydrogel-based patches represent another innovation in TDDS, offering enhanced dermal adhesion, moisture retention, and biocompatibility. These systems can sustain drug release over extended periods while minimizing irritation and improving patient comfort. Hydrogel matrices can also be engineered to respond to temperature or pH shifts, enabling stimulus-sensitive release aligned with circadian symptom fluctuations in conditions such as bipolar disorder or insomnia [40,41].

TDDSs confer distinct safety advantages in neuropsychiatric pharmacotherapy. By avoiding hepatic metabolism, they reduce the formation of hepatotoxic or reactive metabolites, a concern with drugs such as venlafaxine or clozapine. Additionally, the minimization of Cmax-associated side effects, such as sedation, orthostatic hypotension, or QT prolongation, may enhance tolerability and adherence, particularly in vulnerable populations such as the elderly or those with comorbid cardiovascular conditions.

From a clinical operations perspective, TDDSs reduce pill burden and offer covert administration options, which are beneficial for patients with paranoia, swallowing difficulties, or stigma-related treatment resistance. Furthermore, in long-term care and correctional facilities, patch-based regimens simplify dosing logistics and reduce the risk of medication diversion.

However, TDDS remain limited to molecules with suitable physicochemical properties and are susceptible to variability due to skin condition, hydration, temperature, and application site. Skin irritation, adhesive dermatitis, and unpredictable absorption profiles in diseased or damaged skin are potential drawbacks. Moreover, patient misuse, such as partial detachment, exposure to heat sources, or unregulated patch replacement, can alter pharmacokinetics and compromise therapeutic efficacy.

In conclusion, transdermal systems represent a viable and increasingly sophisticated modality for the systemic delivery of neuropsychiatric agents. With the integration of microneedles, hydrogel technologies, and controlled-release matrices, TDDS can improve bioavailability, mitigate systemic toxicity, and enhance adherence, particularly in populations requiring long-term pharmacologic stabilization.

Prodrug Strategies

Prodrug strategies involve the chemical derivatization of active pharmaceutical ingredients (APIs) into pharmacologically inert precursors that undergo biotransformation in vivo to release the active moiety. This approach is employed to optimize physicochemical properties, improve membrane permeability, extend half-life, and mitigate metabolic liabilities, thereby enhancing both pharmacokinetic (PK) and pharmacodynamic (PD) performance. In neuropsychiatric pharmacotherapy, prodrugs offer a rational means of circumventing barriers such as low oral bioavailability, extensive first-pass metabolism, and high susceptibility to cytochrome P450-mediated drug-drug interactions (DDIs) [42].

The design of a successful prodrug hinges on the identification of enzymatic or chemical triggers - typically esterases, amidases, or reductases - that are ubiquitous, predictable, and selectively expressed at the site of drug action. By masking polar functional groups or enhancing lipophilicity, prodrugs can facilitate gastrointestinal absorption, BBB penetration, or lymphatic uptake. Moreover, site-specific activation strategies can minimize off-target effects, reduce systemic toxicity, and extend therapeutic exposure profiles.

A prototypical example is lisdexamfetamine dimesylate, a lysine-conjugated prodrug of dextroamphetamine approved for attention-deficit/hyperactivity disorder (ADHD). This molecule undergoes enzymatic cleavage by red blood cell amidases, yielding a delayed and gradual release of the active stimulant. The rate-limited bioconversion imparts a smoother PK profile, reducing abuse potential, rebound effects, and plasma concentration fluctuations associated with immediate-release formulations [43].

Prodrugs can also decouple therapeutic activity from variable hepatic metabolism, an advantage in patient populations with polymorphisms in CYP2D6, CYP3A4, or CYP2C19. For instance, some investigational CNS-targeted prodrugs are being engineered to bypass hepatic activation pathways entirely, instead relying on CNS-specific enzymatic activation (e.g., β-glucuronidases or proteases), thereby reducing interindividual variability and enhancing therapeutic predictability [44].

Moreover, prodrugs can be co-optimized with transport-mediated uptake pathways to enhance CNS exposure. Examples include L-type amino acid transporter 1 (LAT1)-targeted prodrugs, which exploit endogenous nutrient transporters at the BBB to facilitate brain-selective drug delivery. This strategy is particularly promising for hydrophilic or anionic drugs that would otherwise demonstrate negligible CNS penetration [45].

Emerging platforms are also investigating dual-action or polypharmacologic prodrugs that simultaneously release multiple active agents or exert multimodal activity upon cleavage. For instance, bifunctional prodrugs combining anti-depressant and anti-inflammatory effects may address the multifactorial pathophysiology of treatment-resistant depression or psychotic depression. Such constructs offer the possibility of synergistic efficacy with reduced pill burden and improved adherence [46].

Furthermore, theranostic prodrugs - engineered to release both a therapeutic agent and an imaging tracer - are under preclinical development to allow concurrent tracking of biodistribution and real-time assessment of drug activation. These systems hold promise for dynamically titrated, image-guided neuropsychiatric therapy, especially in clinical trials or highly individualized care pathways.

However, prodrug design entails challenges such as incomplete or variable bioconversion, toxicity of linker groups or byproducts, and complex synthesis routes that may hinder scalability. Regulatory requirements for both the parent compound and the active metabolite further complicate clinical translation, especially when novel metabolic pathways are involved.

In summary, prodrug strategies offer a rational and flexible approach to overcoming key pharmacokinetic and safety limitations in neuropsychiatric drug therapy. By enabling controlled activation, metabolic bypass, and targeted delivery, prodrugs can improve therapeutic precision, reduce DDI liability, and enhance patient-centric outcomes across a spectrum of mental health disorders.

Other Emerging DDS

In addition to nanocarriers, transdermal platforms, and prodrug strategies, several advanced drug delivery systems (DDS) are being developed to address challenges associated with sustained drug exposure, adherence, and individualized therapy in neuropsychiatric care. These include depot injectables, implantable systems, smart DDS, neuroprosthetic-integrated platforms, and theranostic constructs. Collectively, these modalities aim to improve long-term treatment outcomes by enabling programmable, continuous, or real-time responsive drug administration.

Depot Injectables

Depot formulations utilize biodegradable polymers or lipophilic carriers to deliver active pharmaceutical ingredients (APIs) via intramuscular or subcutaneous injection over extended durations, ranging from weeks to months. These long-acting injectables (LAIs) reduce dosing frequency and improve adherence-key factors in the management of chronic psychiatric conditions such as schizophrenia and bipolar disorder. Aripiprazole lauroxil, a long-acting prodrug of aripiprazole, exemplifies this approach, enabling sustained therapeutic plasma concentrations for up to eight weeks post-injection. Other agents, including risperidone and paliperidone palmitate, have demonstrated similar clinical benefits, including reduced relapse rates and improved continuity of care [47]. Despite their utility, depot systems may present limitations including injection-site reactions, inflexible dose titration, and the need for extended pharmacovigilance due to their prolonged activity.

Implantable Delivery Systems

Implantable DDS provides continuous or programmable drug release through subcutaneous or intrathecal devices. These systems can deliver therapeutics over months or years, depending on formulation kinetics and device design. For example, experimental lithium implants have been proposed to address adherence issues in bipolar disorder by maintaining stable serum concentrations while avoiding systemic peaks that contribute to toxicity. Subdermal anti-psychotic implants, such as extended-release risperidone, are under clinical investigation and have shown promising pharmacokinetic profiles, including reduced plasma fluctuation and improved time in therapeutic range [48]. However, surgical insertion, retrieval requirements (for non-biodegradable systems), and risk of infection remain significant considerations.

 Smart Drug Delivery Systems

Smart DDS incorporates sensors, microprocessors, or bioresponsive materials capable of modulating drug release in response to physiological cues or external stimuli. These platforms enable closed-loop, adaptive therapy, aligning drug administration with dynamic biological feedback. The most clinically advanced example is Abilify MyCite, an oral aripiprazole tablet embedded with an ingestible sensor that communicates ingestion events to a wearable patch and smartphone interface. While it does not control drug release, it provides real-time adherence monitoring, supporting medication management in populations with poor insight or high non-adherence risk. Experimental systems include hydrogels that release antidepressants in response to inflammatory cytokines or circadian gene expression patterns, and microneedle-based sensors coupled with electrochemical detectors for stimulus-triggered release. These technologies offer the potential for highly individualized therapy but remain in early development [49].

Neuroprosthetic-Integrated DDS

Neuroprosthetic platforms, including brain-computer interfaces (BCIs) such as Neuralink, represent a speculative but potentially transformative innovation in neuroadaptive drug delivery. These devices are designed to both monitor neural activity and stimulate specific brain regions, with future iterations anticipated to integrate drug reservoirs for closed-loop, neuroresponsive pharmacotherapy. In this envisioned model, intracranial sensors would detect biomarkers of disease activity, such as aberrant firing in dopaminergic circuits, triggering the localized release of anti-psychotic or neuromodulatory agents from microfluidic channels or electrochemically actuated implants. While no such systems have yet demonstrated drug release capabilities in humans, preclinical studies in rodents suggest the feasibility of real-time neurosignal-guided dosing. Major barriers to clinical translation include ethical considerations, neurosurgical risk, immunoreactivity, and the lack of regulatory frameworks for combined neuroelectronic-pharmacological systems. Nonetheless, BCIs represent a potential paradigm shift in the treatment of refractory neuropsychiatric conditions [50].

Theranostic DDS

Theranostic DDS co-delivers therapeutic agents and diagnostic tools, such as imaging tracers, biosensors, or signal reporters, enabling simultaneous drug administration and monitoring. These systems are particularly relevant for personalized psychiatry, where objective biomarkers of treatment response remain limited. For example, nanoparticles co-loaded with anti-depressants and fluorescent dyes have been used to visualize BBB penetration and assess real-time CNS drug accumulation in preclinical models. Other constructs incorporate PET or MRI contrast agents, allowing non-invasive imaging of drug biodistribution, release kinetics, and receptor occupancy. Theranostic systems facilitate adaptive dosing, early identification of non-responders, and mechanistic insights into drug action, positioning them as valuable adjuncts in both research and clinical contexts. However, complexity in design, cost of production, and regulatory classification continue to restrict widespread clinical application. Table 1 highlights the different drug delivery systems (DDSs), their mechanisms, advantages, and potential disadvantages.

**Table 1:** Summary of drug delivery systems (DDSs), highlighting their mechanisms, advantages, and potential disadvantages. The table is created by the author of this study.

Type of DDS	Mechanism	Advantages	Disadvantages
Nanotechnology-based DDS	Utilizes nanoparticles (liposomes, micelles, dendrimers) for targeted delivery across biological barriers.	Enhanced bioavailability, CNS targeting, reduced side effects, controlled release.	High development cost, complex manufacturing, and potential long-term toxicity concerns.
Transdermal systems	Delivers drugs through the skin, bypassing first-pass metabolism.	Steady plasma drug levels, improved compliance, reduced GI side effects.	Limited to drugs with specific properties (e.g., low molecular weight), potential for skin irritation.
Prodrug formulations	Converts inactive precursors into active drugs via enzymatic or chemical transformation.	Reduced drug-drug interactions, improved stability, and lower abuse potential.	Requires precise enzymatic conditions, delayed onset of action, not suitable for all drug types.
Depot injections	Long-acting formulations injected subcutaneously or intramuscularly.	Prolonged release, improved adherence, and reduced dosing frequency.	Pain or discomfort at injection site, difficulty in dose adjustment once administered.
Implantable systems	Implants that release drugs at a controlled rate over extended periods.	Long-term delivery, minimizes non-adherence, and consistent plasma levels.	Surgical implantation required, risk of infection, expensive.
Smart drug delivery systems	DDS integrated with sensors or responsive materials that adapt to physiological conditions.	Real-time monitoring, precise dosing, and reduced side effects.	High cost, complex technology, and regulatory challenges.
Oral extended-release systems	Utilizes matrix systems or coatings to release drugs gradually in the gastrointestinal tract.	Reduced dosing frequency, improved patient adherence, and steady therapeutic levels.	Potential for dose dumping, not suitable for drugs with narrow therapeutic windows.
Nanogels and theranostics	Combines therapy and diagnostics, delivering drugs and enabling real-time imaging or monitoring.	Simultaneous treatment and monitoring, reduced off-target effects.	Expensive to develop, regulatory hurdles, and limited real-world applications to date.

Clinical implications of DDS

Efficacy Improvements

Advanced drug delivery systems (DDSs) substantially enhance therapeutic efficacy in neuropsychiatric treatment by overcoming conventional pharmacokinetic (PK) constraints and optimizing pharmacodynamic (PD) target engagement. Traditional oral and parenteral formulations are often limited by variable absorption, hepatic first-pass metabolism, low blood-brain barrier (BBB) permeability, and erratic plasma concentration-time profiles. DDSs mitigate these issues by improving bioavailability, enhancing CNS selectivity, and sustaining therapeutic exposure, resulting in improved symptom control, reduced relapse rates, and broader treatment responsiveness across heterogeneous patient populations.

Nanoparticle-based DDS, particularly liposomes, polymeric micelles, and solid lipid nanoparticles, enable preferential CNS accumulation through mechanisms such as receptor-mediated transcytosis or adsorptive-mediated transport. These carriers shield the active pharmaceutical ingredient (API) from enzymatic degradation and facilitate controlled, site-specific release. In preclinical models of schizophrenia and depression, nanoparticle formulations of risperidone, fluoxetine, and venlafaxine have demonstrated superior therapeutic efficacy relative to conventional formulations, as evidenced by greater behavioral normalization, higher CNS drug concentrations, and extended duration of action [51].

Transdermal and depot systems also contribute to efficacy by providing continuous drug delivery, thereby eliminating peak-trough oscillations commonly associated with oral dosing. These fluctuations are a key contributor to therapeutic failure and side effects, particularly in agents with narrow therapeutic windows. For example, long-acting injectable (LAI) anti-psychotics such as paliperidone palmitate and aripiprazole lauroxil maintain steady plasma concentrations over weeks or months, reducing the risk of symptom breakthrough, hospitalization, and caregiver burden [52]. Meta-analyses of LAI use in schizophrenia have shown significant improvements in medication adherence, symptom remission, and relapse prevention compared to oral anti-psychotics [53].

Moreover, DDSs facilitate the implementation of multidrug strategies within a single construct, enabling synergistic mechanisms of action and expanding the therapeutic landscape. Multifunctional nanocarriers have been designed to co-encapsulate anti-depressants and anti-inflammatory agents, or anti-psychotics with cognitive enhancers, offering a unified treatment modality for complex psychiatric presentations. Such platforms allow for synchronized release kinetics, optimized ratio control, and reduced pill burden, each of which contributes to more robust clinical efficacy in polypharmacy-dependent disorders such as bipolar disorder or schizoaffective disorder.

Innovations in DDS have also advanced treatment options for patients with treatment-resistant depression (TRD), a condition often refractory to conventional oral therapies. Controlled-release intranasal, intrathecal, and nanocarrier-enhanced formulations of ketamine and esketamine have shown superior anti-depressant effects and faster onset of action compared to oral or intravenous routes, likely due to more efficient CNS targeting and sustained receptor engagement. These systems enhance NMDA receptor modulation and downstream synaptogenesis while reducing dissociative and cardiovascular side effects associated with bolus administration.

Furthermore, DDSs reduce the pharmacokinetic variability caused by individual differences in metabolism, gut motility, and genetic polymorphisms in cytochrome P450 enzymes. This results in more consistent drug exposure and therapeutic outcomes, particularly when integrated with pharmacogenomic-guided dosing strategies. For instance, depot or prodrug formulations of CYP2D6-metabolized anti-psychotics can bypass the variable hepatic enzyme activity observed in poor or ultrarapid metabolizers, providing a more predictable therapeutic response.

In summary, DDSs enhance neuropsychiatric drug efficacy through a multifaceted mechanism - improving CNS bioavailability, sustaining drug exposure, enabling multidrug co-delivery, bypassing metabolic liabilities, and reducing interindividual variability. These properties collectively improve symptom control, reduce relapse frequency, and expand treatment options for complex and refractory psychiatric disorders.

Safety Enhancements

The safety profiles of psychotropic medications can be significantly enhanced through the application of advanced drug delivery systems (DDSs). These technologies reduce systemic drug exposure, thereby decreasing the incidence of adverse effects and improving the therapeutic index of neuropsychiatric agents. A key advantage of DDS is their capacity for targeted delivery, enabling site-specific drug accumulation while limiting distribution to non-target tissues. This spatial precision is particularly valuable in psychopharmacology, where systemic side effects, such as sedation, metabolic disturbances, or cardiovascular complications, are often dose-limiting and exacerbated in agents with narrow therapeutic windows. For example, nanoparticles engineered to cross the blood-brain barrier (BBB) offer a prospective therapeutic advantage - supported by preliminary evidence - by facilitating selective central nervous system (CNS) delivery of anti-psychotics or anti-depressants, thereby potentially reducing the peripheral side effects commonly associated with conventional oral or parenteral formulations.

Prodrug strategies also contribute to improved safety by minimizing metabolic overload and reducing the risk of drug-drug interactions (DDIs) - a strategic advantage of prodrug design with both demonstrated and theoretical relevance in lessening metabolic burden and interaction potential. These pharmacologically inert precursors are designed to undergo biotransformation in vivo, releasing the active drug in a controlled and site-specific manner. By bypassing hepatic cytochrome P450-dependent metabolism - often implicated in DDIs and metabolic byproduct formation - prodrugs reduce the burden on detoxification pathways and enhance safety in polypharmacy contexts. Enzyme-specific or physiologically responsive activation mechanisms ensure that the active drug is liberated only at the desired site or under defined conditions, thereby lowering the likelihood of systemic toxicity and unintended pharmacological effects.

Transdermal DDSs offer another important safety advantage by circumventing gastrointestinal absorption and hepatic first-pass metabolism. This route reduces the risk of gastrointestinal irritation and hepatotoxicity, two common complications associated with oral psychotropic agents. Transdermal patches for anti-psychotics or mood stabilizers provide steady-state drug release through the dermis, minimizing fluctuations in plasma concentration and avoiding peaks that may trigger dose-dependent adverse effects. These systems are particularly beneficial for patients with hepatic impairment or those on complex polypharmacy regimens, as they reduce hepatic enzyme exposure and improve overall tolerability.

Smart DDS, which are engineered to release their payloads in response to specific physiological triggers, add a further layer of safety by restricting drug activation to pathologically relevant conditions. These systems may be designed to respond to local pH shifts, redox gradients, enzymatic activity, or inflammatory biomarkers, thereby ensuring drug release occurs only when therapeutically necessary. In psychiatric applications, such precision is essential for maintaining stable drug levels while avoiding overshooting therapeutic thresholds that could lead to sedation, agitation, or toxic effects. By offering self-regulating release kinetics, smart DDSs reduce the risk of overdose and mitigate adverse interactions, particularly in patients with erratic adherence or fluctuating metabolic states [54].

Populations with comorbidities, such as the elderly or those with cardiovascular, hepatic, or renal dysfunction, derive particular benefit from DDS that limit systemic exposure. These individuals often exhibit altered pharmacokinetics and are more susceptible to drug accumulation and organ-specific toxicities. DDS technologies such as nanoparticle-mediated CNS targeting and transdermal formulations minimize the pharmacological burden on compromised organ systems. In addition, CNS-activated prodrugs that release the active compound selectively within the brain offer a promising approach to avoiding peripheral side effects, including those affecting the heart, liver, or kidneys. Such targeted prodrugs enhance both efficacy and safety, particularly in vulnerable patient groups requiring long-term pharmacologic maintenance.

By enabling targeted delivery, physiologically responsive activation, and controlled release, DDSs significantly improve the safety landscape of neuropsychiatric treatment. These innovations support the safer use of psychotropic agents in patients with chronic mental illness, multiple comorbidities, or heightened sensitivity to systemic drug exposure. Ultimately, DDSs not only enhance therapeutic efficacy but also broaden the safety margin, facilitating more individualized and tolerable psychiatric care.

Patient Adherence

Drug delivery systems (DDSs) have a substantial impact on improving patient adherence by mitigating several key barriers to compliance, including complex dosing regimens, adverse side effects, and the inconvenience associated with frequent medication administration. One of the primary advantages of DDSs is their capacity to simplify pharmacotherapeutic schedules, thereby promoting consistent medication use, particularly critical in the long-term management of chronic neuropsychiatric conditions. By providing extended-release formulations, transdermal systems, and depot injections, DDSs significantly reduce dosing frequency, enabling the maintenance of therapeutic drug levels with fewer daily interventions. This not only alleviates the cognitive and logistical burden on patients but also enhances satisfaction with treatment, especially among populations with cognitive decline, psychiatric comorbidities, or functional impairments such as older adults and individuals with neurological disorders.

DDSs also improve adherence by enhancing tolerability and reducing the incidence of side effects, which are leading causes of non-compliance in psychopharmacology. Many agents used to manage schizophrenia, bipolar disorder, and major depression are associated with undesirable effects such as sedation, weight gain, and metabolic disturbances. DDSs mitigate these adverse effects by enabling more consistent and targeted drug release, thereby avoiding the pharmacokinetic peaks and troughs that can trigger acute toxicity or symptom rebound. For example, transdermal and depot formulations provide a controlled release profile that minimizes peripheral drug exposure, thereby reducing the likelihood of systemic adverse events and improving overall treatment acceptability.

The integration of digital technologies with DDS has further enhanced adherence by offering real-time monitoring capabilities and patient support mechanisms. Sensor-enabled platforms, such as ingestible event markers or wearable patches, can track drug ingestion and transmit adherence data to healthcare providers, enabling timely intervention in cases of missed doses or non-compliance. These systems promote a collaborative model of care by fostering communication between patients and providers and facilitating personalized adjustments to treatment regimens. Real-time adherence tracking not only empowers patients but also improves therapeutic continuity, reduces relapse risk, and enhances the precision of clinical decision-making.

For populations with high non-adherence risk, such as individuals with ADHD or schizophrenia, DDSs provide critical tools for treatment stabilization. In these conditions, patients may exhibit poor insight, forgetfulness, or intentional avoidance of medication, all of which can undermine therapeutic efficacy. Extended-release formulations help sustain plasma drug levels throughout the day, reducing the need for multiple daily doses and minimizing symptom fluctuations. Depot injectables further address this challenge by eliminating the need for daily administration altogether, ensuring sustained treatment coverage over weeks or months. These formulations have demonstrated significant improvements in treatment persistence and relapse prevention in patients with chronic psychiatric illness.

Furthermore, novel DDSs such as microneedle patches offer non-invasive, painless drug administration and are particularly advantageous for patients with needle phobia or swallowing difficulties. These systems deliver medications transdermally through microscopic projections that penetrate the stratum corneum without stimulating pain receptors. Microneedle DDS is an emerging technology with potential applicability to psychiatric treatment; while clinical validation is still needed, they represent a promising approach for improving the overall treatment experience and expanding therapeutic accessibility, particularly for individuals who may resist conventional oral or injectable formulations. By offering a more patient-centric mode of administration, these technologies support broader engagement in pharmacologic care and reinforce long-term adherence.

Overall, DDSs provide an integrated and multifaceted solution to the persistent challenge of non-adherence in neuropsychiatric care. Through simplified regimens, improved tolerability, real-time monitoring, and non-invasive delivery options, these systems enhance both the feasibility and appeal of chronic pharmacologic treatment. As illustrated in Table 2, the application of DDS across a range of psychotropic agents has led to demonstrable improvements in patient adherence and therapeutic outcomes.

**Table 2:** Examples of drug delivery systems (DDSs) in neuropsychiatric treatments. PTSD: post-traumatic stress disorder; ADHD: attention-deficit/hyperactivity disorder The table is created by the author of this study.

Drug/medication	Trade name	Delivery system	Condition treated	Benefits
Risperidone	Risperdal	Liposomal nanoparticles	Schizophrenia	Enhanced brain targeting, reduced side effects
Selegiline	Emsam	Transdermal patch	Depression, Parkinson’s	Bypasses first-pass metabolism
Lisdexamfetamine	Vyvanse	Prodrug formulation	ADHD	Reduced abuse potential, steady release
Aripiprazole lauroxil	Aristada	Depot injection	Schizophrenia, bipolar disorder	Sustained release, improved adherence
Lithium	Lithium carbonate	Implantable pump	Bipolar disorder	Controlled, long-term drug release
Escitalopram	Lexapro	Polymeric micelles	Depression, anxiety	Improved solubility and bioavailability
Clozapine	Clozaril	Stimuli-responsive nanoparticles	Schizophrenia	Precise, on-demand drug release
Olanzapine	Zyprexa	Nanogel	Schizophrenia	Reduced systemic exposure, enhanced targeting
Methylphenidate	Concerta	Hydrogel-based transdermal patch	ADHD	Extended release, improved compliance
Venlafaxine	Effexor XR	Solid lipid nanoparticles	Major depressive disorder	Increased bioavailability, reduced dosing frequency
Duloxetine	Cymbalta	Nanocrystal formulation	Generalized anxiety disorder	Enhanced dissolution, faster onset of action
Quetiapine	Seroquel XR	Oral extended-release tablets	Bipolar disorder, schizophrenia	Reduced dosing frequency, improved adherence
Haloperidol	Haldol	Depot injection	Psychosis, schizophrenia	Sustained release, consistent plasma levels
Sertraline	Zoloft	Transdermal patch	Depression, PTSD	Improved patient compliance, steady drug levels
Bupropion	Wellbutrin SR	Extended-release formulation	Depression, smoking cessation	Minimized withdrawal symptoms, steady effect
Aripiprazole	Abilify MyCite	Oral tablet + digital sensor (smart DDS)	Schizophrenia, bipolar disorder	Real-time adherence monitoring

Integration With Personalized Medicine

The future of neuropsychiatric pharmacotherapy is increasingly aligned with the principles of personalized medicine - a paradigm that seeks to tailor treatment regimens to the individual patient’s genetic makeup, biomarker profile, metabolic phenotype, and environmental context. In this evolving therapeutic landscape, advanced drug delivery systems (DDSs) are poised to play a transformative role by enabling precision-targeted interventions. These systems are designed to deliver the right drug, at the optimal dose, to the intended site of action, and at the appropriate time, thereby improving therapeutic efficacy and minimizing adverse outcomes.

Genetic polymorphisms in drug-metabolizing enzymes, particularly CYP2D6, CYP2C19, and CYP3A4, are a major source of interindividual variability in psychotropic drug response. Patients classified as poor metabolizers of CYP2D6 substrates, such as risperidone, fluoxetine, or aripiprazole, may exhibit exaggerated drug exposure and associated toxicity, while ultrarapid metabolizers may fail to achieve therapeutic plasma levels, resulting in suboptimal response or clinical relapse [55]. DDS can mitigate the impact of such genetic variability through alternative activation or absorption pathways. Some prodrugs bypass hepatic metabolism, offering improved safety in variable metabolizers; this is discussed further below. Novel prodrug candidates are now being designed to leverage non-CYP enzymatic activation, further reducing dependency on polymorphic hepatic pathways. Similarly, nanoparticles and micellar DDS can bypass hepatic first-pass metabolism by targeting the lymphatic system or delivering drugs directly across the blood-brain barrier (BBB) [56-59].

The incorporation of biomarker-guided strategies into DDS design is another major advancement in personalized psychiatry. Biomarkers reflecting disease activity, pharmacodynamic response, or pathophysiologic states - such as neuroinflammation, oxidative stress, or altered CNS pH - can be used to guide drug release. DDSs engineered to respond selectively to such biomarker-defined microenvironments offer a platform for highly localized and conditional drug delivery. Stimuli-responsive nanoparticles exemplify this approach, releasing their therapeutic payloads in response to local cues such as acidic pH, elevated enzymatic activity, or redox imbalances. For example, pH-sensitive micelles have been investigated for the targeted delivery of anti-depressants in acidic brain regions associated with depressive pathology. In parallel, aptamer-functionalized nanoparticles targeting disease-relevant proteins such as the transferrin receptor or amyloid aggregates are being explored to enhance brain-specific targeting, offering new opportunities for multi-modal and multi-targeted DDS interventions.

The complexity of modern DDS design has also driven the integration of artificial intelligence (AI) and machine learning (ML) as essential tools in formulation development and optimization. ML algorithms have been used to predict drug-excipient compatibility based on molecular descriptors, estimate BBB permeability via molecular docking and quantitative structure-activity relationship (QSAR) models, and identify optimal DDS modalities for specific psychiatric indications using large-scale clinical and pharmacogenomic datasets. AI is also being applied to predict drug release kinetics, scale-up parameters, and subgroup-specific DDS performance, facilitating the design of depot versus oral systems tailored to individual patient characteristics. Moreover, virtual clinical trials using AI-simulated patient models allow for the preclinical evaluation of DDS performance across genetic and demographic variability, reducing development time and cost [60-64].

Theranostics, the convergence of therapeutic and diagnostic modalities, represents a promising frontier in the development of personalized DDS. Smart DDS platforms incorporating embedded sensors are under investigation for real-time monitoring of drug release events, adherence patterns, and local biomarkers such as pH or enzymatic activity. These systems can relay data via wearable or mobile interfaces, enabling dynamic treatment adjustments. One approved example is Abilify MyCite, a sensor-integrated aripiprazole tablet that transmits ingestion data to a digital platform, facilitating adherence monitoring in schizophrenia and bipolar disorder. More advanced systems under development include implantable drug reservoirs paired with neurotransmitter biosensors, which adjust dosing in real time based on brain levels of dopamine or serotonin.

Nanotheranostic systems further expand this potential by delivering therapeutic agents alongside imaging tracers, enabling simultaneous drug administration and visualization of pharmacologic distribution. These platforms are particularly valuable in psychiatric drug development, where objective biomarkers of treatment response remain limited. The ability to titrate dosing based on real-time neuroimaging data offers the potential for truly adaptive therapy, dynamically matched to patient neurobiology. Table 3 compares advanced drug delivery systems (DDSs) based on efficacy in neuropsychiatric disorders, cost, patient acceptability, and developmental stage.

**Table 3:** Comparative evaluation of advanced drug delivery systems (DDSs) based on efficacy in neuropsychiatric disorders, cost, patient acceptability, and developmental stage. ADHD: attention-deficit/hyperactivity disorder; MDD: major depressive disorder; CNS: central nervous system The table is created by the author of this study.

DDS type	Efficacy in neuropsychiatric disorders	Cost	Patient acceptability	Developmental stage
Nanotechnology-based	High (preclinical success; CNS targeting)	High	Moderate	Preclinical to phase I/II
Transdermal systems	Moderate (e.g., selegiline for MDD)	Moderate	High	Marketed (for select drugs)
Prodrug formulations	High (e.g., lisdexamfetamine in ADHD)	Moderate	High	Marketed/under development
Depot injections	High (e.g., aripiprazole lauroxil)	Moderate	Moderate	Marketed (widespread use)
Implantable systems	Potentially high (experimental)	High	Low	Early-stage research
Smart DDS	Emerging, promising for adherence/monitoring	Very high	Variable	Developmental/pilot use
Oral ER formulations	Moderate to high (e.g., Paliperidone ER)	Low to moderate	High	Marketed
Nanogels/theranostics	Promising (multi-drug, diagnostic use)	High	Moderate	Preclinical/conceptual

Challenges and future directions

**Figure 2:**
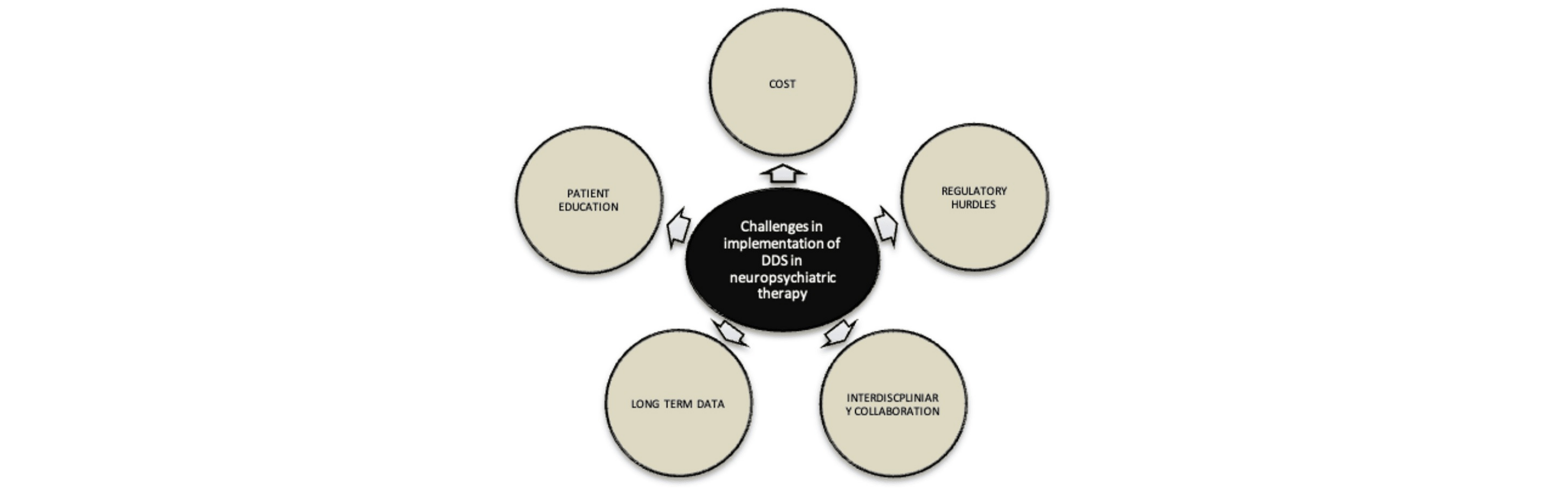
Challenges associated with implementation of DDSs in neuropsychiatric therapy. DDSs: drug delivery systems The figure is created by the author of this study.

Despite the significant benefits demonstrated by drug delivery systems (DDSs) in improving therapeutic outcomes, several challenges must be addressed for their widespread implementation. These obstacles include cost, regulatory hurdles, patient education, the need for long-term data, and the requirement for interdisciplinary collaboration (Figure 2).

Cost and Accessibility

Despite their significant therapeutic benefits, advanced drug delivery systems (DDSs) often face limited global accessibility due to their high development, production, and distribution costs. These economic barriers hinder the widespread adoption of DDS technologies, especially in low- and middle-income countries (LMICs), where resource constraints, infrastructure limitations, and reimbursement challenges further complicate access.

Economic Barriers to Widespread Adoption

Advanced DDS, such as nanocarriers, implantables, depot formulations, and smart systems, often require specialized excipients, precision engineering, and complex manufacturing protocols, driving up production costs. For example, the fabrication of liposomal or polymeric nanoparticles involves high-purity solvents, sterility controls, and stringent quality assurance, contributing to elevated per-unit costs. Similarly, implantable systems demand surgical placement, biocompatible materials, and long-term release validation, all of which increase the total cost of care [65]. These high expenses are compounded by regulatory compliance costs, intellectual property licensing, cold-chain logistics (for biologics-based DDS), and the low scalability of many novel formulations. As a result, many of these technologies are limited to high-income markets and specialty care centers, leaving large populations without access to potentially life-improving treatments.

Strategic Approaches to Improve Affordability

Addressing the cost-access gap in DDS development requires a multifaceted approach. Streamlined manufacturing processes, such as continuous processing, microfluidics, and spray-drying-based nanocarrier production, can reduce batch variability and lower per-unit costs. Material substitution is another viable strategy, involving the use of low-cost, biodegradable polymers or naturally derived carriers like chitosan and starch-based materials in place of synthetic or proprietary systems. Furthermore, scalable device engineering, including the development of modular and reusable components such as smart patches or refillable pumps, can significantly cut down on disposable material usage. In parallel, promoting open-access design frameworks and fostering technology transfer initiatives may empower manufacturers in low- and middle-income countries (LMICs) to replicate advanced DDS platforms more affordably, provided they align with local regulatory requirements.

Role of Public-Private Partnerships and Policy Support

Public-private partnerships (PPPs) can play a pivotal role in accelerating the affordability and distribution of advanced DDS, particularly for priority health conditions such as schizophrenia, epilepsy, and treatment-resistant depression. Government-subsidized development programs can support the creation of long-acting injectables or transdermal systems tailored to underserved populations. Non-profit consortia, such as PATH and the Medicines Patent Pool, facilitate licensing and enable generic manufacturing of mature DDS platforms. Additionally, tiered pricing models backed by international health agencies help ensure equitable cost-sharing between high- and low-income markets. By supporting policies that further strengthen these efforts, governments can promote affordable innovation through mechanisms such as tax credits, orphan drug exclusivity extensions, or advance market commitments, especially in pediatric and psychiatric care, where DDSs can significantly enhance treatment adherence and therapeutic outcomes.

Future Research Directions

To sustainably improve DDS accessibility, future research should prioritize several key areas. Low-cost DDS prototyping is essential, with studies needed to compare cost-to-outcome ratios between polymer-based nanocarriers and lipid-based systems in treating conditions like schizophrenia or bipolar disorder. Decentralized manufacturing models should be explored through the development of portable, small-scale DDS production systems utilizing technologies such as 3D printing or modular assembly, enabling on-site generation in remote or resource-limited settings. Research into biodegradable implant technologies is also critical, particularly for self-dissolving subcutaneous implants that eliminate the need for retrieval surgery and reduce healthcare resource consumption. Open-source DDS platforms represent another promising direction, with collaborative efforts between academic institutions and start-ups focused on publishing publicly accessible blueprints for DDS materials, designs, and assembly processes, thereby encouraging frugal innovation. Additionally, behavioral economics trials should be conducted to evaluate how pricing models, co-payment reductions, and integration with mobile health platforms affect patient uptake and long-term adherence to DDS therapies. In parallel, expanding cost-effectiveness studies using metrics such as quality-adjusted life years (QALY) and disability-adjusted life years (DALY) will be essential to support the incorporation of DDS into universal health coverage schemes and national formularies.

Regulatory Hurdles in the Development and Approval of Advanced Drug Delivery Systems

The regulatory landscape for advanced drug delivery systems (DDS) presents significant challenges due to the inherent complexity of these platforms. Unlike conventional dosage forms, DDSs often involve novel materials, device-drug combinations, or stimuli-responsive mechanisms that fall outside the scope of existing regulatory paradigms. This section outlines key regulatory pathways, clinical trial requirements, and harmonization issues that affect the development and global accessibility of DDS technologies, particularly in the context of neuropsychiatric therapeutics.

Regulatory Pathways and Classification Complexities

The regulatory classification of DDS is often ambiguous, particularly when the product combines elements of both drug and device. In the United States, the Food and Drug Administration (FDA) evaluates DDS under different pathways depending on the product's primary mode of action (PMOA). DDS may be regulated as drugs, devices, biologics, or combination products, coordinated by the Office of Combination Products (OCP) [66]. Common approval pathways include 505(b)(1) for novel drug entities and delivery systems requiring full safety and efficacy data, 505(b)(2) for previously approved drugs with new delivery platforms allowing reliance on existing clinical data, and 510(k) or PMA for device-led systems such as transdermal patches, implants, or microneedle-based delivery. An Investigational Device Exemption (IDE) may also be required when the delivery component is novel and evaluated separately from the active pharmaceutical ingredient (API) [67]. The classification of emerging technologies such as nanocarriers, theranostics, or digital DDS platforms remains particularly challenging, as these systems may straddle multiple regulatory categories and often require input from the Center for Drug Evaluation and Research (CDER), the Center for Devices and Radiological Health (CDRH), and sometimes the Center for Biologics Evaluation and Research (CBER). This regulatory ambiguity can result in inconsistent guidance, extended review timelines, and increased development costs [68].

Preclinical and Clinical Requirements

Regulatory approval of DDS demands comprehensive characterization of both the active drug and the delivery system. Preclinical evaluation typically includes pharmacokinetic and toxicokinetic studies across species to assess absorption, metabolism, biodistribution, and excretion; tissue compatibility and immunogenicity testing, particularly for depot formulations, implants, and nanoparticles; and mechanical integrity and performance assessments for device components, including adhesion for transdermal systems, degradation for implants, and trigger sensitivity for smart DDSs [69]. Clinical development phases are similarly complex. In addition to standard safety and efficacy endpoints, sponsors must often demonstrate bioequivalence to existing therapies, particularly under the 505(b)(2) pathway, device usability studies including human factors testing for wearable or implantable DDS, in-use tolerability and application-site safety for transdermal or subcutaneous systems, and real-world performance in adherence, safety, and pharmacodynamic response, particularly for digital DDS. For instance, long-acting injectable formulations such as aripiprazole lauroxil require detailed modeling of drug release over a four- to eight-week dosing period, as well as post-marketing studies to monitor relapse prevention and patient adherence [70]. Similarly, nanoparticle-based DDS must demonstrate not only consistent API release profiles but also long-term clearance and safety in vivo [71].

International Harmonization and Access Disparities

A significant barrier to global access to DDS technologies lies in the lack of regulatory harmonization. While the International Council for Harmonisation (ICH) has developed unified guidelines for general drug development, such as ICH M4, E6, and M9, harmonized approaches specific to complex delivery systems remain limited. The European Medicines Agency (EMA) has issued a reflection paper on nanomedicines, recommending case-by-case evaluations of physicochemical characterization, bioavailability, and immunotoxicity, but these remain non-binding, leading to inconsistent implementation across EU member states [72]. In contrast, Japan’s Pharmaceuticals and Medical Devices Agency (PMDA) often requires localized biocompatibility and usability studies even when global data are available [73]. China’s National Medical Products Administration (NMPA) has adopted select ICH standards but demands extensive in vitro data on device performance, particularly for implantables and transdermal systems [74]. These regional inconsistencies delay multinational product launches, discourage investment in global trials, and contribute to therapeutic access inequities, particularly in low- and middle-income countries.

Emerging Solutions and Future Directions

To address these barriers, regulatory bodies have initiated several measures to foster innovation in DDS development. The FDA’s Nanotechnology Regulatory Science Research Plan supports the development of analytical tools and risk-based frameworks for nanomedicine evaluation [75]. The EMA Innovation Task Force (ITF) offers early-stage scientific advice on novel DDS to improve regulatory predictability [76]. The proposed ICH M13 guideline on bioequivalence for complex generics may streamline comparative studies for extended-release or transdermal systems [77]. In parallel, there is growing support for adaptive regulatory models, including conditional approvals based on early efficacy and surrogate endpoints, real-world evidence integration for monitoring long-acting and personalized DDS, and digital biomarker validation, particularly relevant for sensor-enabled or AI-guided DDS platforms. Establishing DDS-specific regulatory frameworks - tailored to delivery type, risk profile, and therapeutic area - will be essential for accelerating innovation and ensuring safe, equitable access to next-generation neuropsychiatric treatments.

Patient Education and Acceptance

The successful implementation of novel drug delivery systems (DDS) extends beyond technological innovation; it also depends on the acceptance, understanding, and compliance of patients and healthcare providers. As DDS platforms become more sophisticated, ranging from transdermal patches and depot injectables to smart pills and implantables, the need for effective education and communication strategies becomes increasingly vital. This is particularly critical in the context of neuropsychiatric disorders, where treatment adherence and trust in therapy significantly impact outcomes [78].

Barriers to Patient Adoption

Patients may exhibit hesitancy or resistance toward adopting novel DDS due to several factors. Familiarity bias often leads to a preference for traditional oral or injectable medications [79], while mistrust of new technologies, especially those involving sensors, implants, or artificial intelligence, can hinder acceptance [80]. Digital literacy limitations, particularly among older adults or socioeconomically disadvantaged groups, also play a role in reduced DDS uptake [81]. Additional concerns include perceived or actual risks related to safety, side effects, and the invasiveness of devices, as well as misconceptions about the cost or accessibility of advanced delivery systems [82]. These barriers are often more pronounced in populations with limited healthcare access or low health literacy, where patients may lack the resources or knowledge to make informed decisions about innovative therapies.

Role of Healthcare Providers and Systems

Healthcare professionals are essential mediators in translating DDS innovation into patient-centered clinical care. To foster successful adoption, clinicians must be adequately trained in the functionality, therapeutic benefits, and limitations of various DDS platforms [83]. They also need to be equipped to communicate both the risks and expectations associated with these systems in a way that is accessible and relatable to diverse patient populations. Furthermore, they should be supported by clear clinical guidelines and decision aids that help identify the most appropriate candidates for specific DDS technologies. A lack of provider education can result in underutilization of effective DDS, inappropriate application in vulnerable populations, or poor patient adherence to recommended therapies [84].

Education Strategies to Enhance Acceptance

Improving patient acceptance of DDS requires culturally sensitive, targeted, and accessible education approaches. Multimedia tools, such as videos, mobile applications, and infographics, can be tailored to different literacy levels and cultural contexts to enhance understanding and engagement [85]. In-clinic demonstrations, especially for physical systems like transdermal patches or depot injectors, offer hands-on exposure that builds comfort and familiarity. Peer-led support groups can also normalize the use of new DDS technologies and help dispel stigma or myths, especially in the management of mental health conditions. Incorporating shared decision-making tools that align patient values and preferences with clinical evidence can further reinforce trust and ownership in treatment decisions. In addition, telemedicine platforms can play a key role in extending educational outreach to remote or underserved regions where in-person engagement may not be feasible [86].

Future Research Directions

To further improve education, acceptance, and adherence to advanced DDS, several research avenues should be pursued. First, behavioral health interventions informed by psychology, such as motivational interviewing or digital nudges, should be designed and tested to improve patient receptivity to new delivery systems [87]. For example, a randomized controlled trial could compare standard versus behavioral coaching-based onboarding in patients prescribed depot anti-psychotics. Second, there is a pressing need to develop digital education tools for low-literacy populations, such as voice-assisted and interactive tutorials in multiple languages for elderly or digitally naïve patients initiating transdermal therapy [88]. Third, standardized and evidence-based provider training modules should be created for pharmacists, nurses, and physicians. These could include immersive formats like virtual reality or simulation-based learning for microneedle patch application and patient counseling [89]. Fourth, mixed-methods studies using surveys, interviews, and focus groups can help map patient perceptions, identify key drivers of DDS acceptance, and clarify route- or condition-specific concerns [90]. A practical example would be a qualitative study exploring attitudes toward implantable versus oral extended-release medications in individuals with bipolar disorder. Lastly, longitudinal adherence studies should assess the impact of structured DDS education programs on therapeutic outcomes. For instance, a cohort study could measure long-term adherence in schizophrenia patients receiving personalized depot injection education versus those under standard care models [91,92].

Long-Term Data and Safety Surveillance of Drug Delivery Systems

While advanced drug delivery systems (DDS) have demonstrated promising outcomes in preclinical and early-phase clinical studies, the lack of long-term safety and efficacy data remains a critical limitation in their widespread adoption. This is especially pertinent for chronic conditions such as schizophrenia, bipolar disorder, ADHD, or major depressive disorder, where treatment durations often span years or even decades. Without robust longitudinal data, clinicians, regulators, and patients may remain cautious about incorporating DDS technologies into standard care.

Importance of Long-Term Evaluation

Most current DDS approvals are based on short- to mid-term clinical trials, typically ranging from several weeks to a year, which are often insufficient to assess sustained therapeutic efficacy, delayed-onset adverse events, or device degradation over time. Long-term use may reveal side effects such as tissue reactions, immunogenic responses, or organ-specific toxicities that are not evident during early evaluations. For biodegradable implants or long-acting injectables, material breakdown and bioresorption may introduce risks like incomplete dissolution or chronic inflammation. Additionally, psychological acceptance and adherence dynamics may shift over prolonged use, particularly in psychiatric populations. For instance, depot anti-psychotic formulations like aripiprazole lauroxil and paliperidone palmitate are gaining popularity for relapse prevention, yet data on their real-world adherence rates and metabolic effects over 5-10 years remain limited. Similarly, nanocarrier-based DDS may raise concerns about tissue accumulation or slow systemic clearance, which could only become apparent after repeated or long-term exposure [93].

Research and Infrastructure Gaps

Several factors contribute to the dearth of long-term data on DDS. One major barrier is the dominance of short funding cycles, which rarely support multi-year follow-up studies. Additionally, post-marketing surveillance frameworks are often underdeveloped or underutilized when it comes to complex DDS technologies. In psychiatric care, high dropout rates and fragmented patient engagement further hinder the continuity of data collection. Limited integration and data-sharing between clinical research centers and healthcare systems exacerbate the challenge, preventing the construction of comprehensive, long-term safety and efficacy profiles that are crucial for broader clinical and regulatory confidence.

Future Research Proposals

To close the long-term data gap, several targeted research strategies are recommended. First, the establishment of global DDS safety registries focused on specific diseases would allow for systematic tracking of patient outcomes over time. For example, a registry dedicated to anti-psychotic DDS could collect data on treatment responses, adverse events, and device tolerability in schizophrenia patients over a 5-10 year period. Such registries would enable meta-analyses across diverse populations and real-world clinical settings. Second, long-term comparative effectiveness trials should be designed to evaluate DDS against conventional therapies in pragmatic, multi-year studies. An illustrative example would be a five-year study comparing relapse rates, metabolic outcomes, and quality of life in bipolar disorder patients treated with oral versus depot DDS formulations of lithium. Third, focused surveillance programs are needed to monitor biodegradable DDS platforms. These should track degradation kinetics, tissue responses, and elimination patterns, such as in the case of subcutaneous biodegradable implants for ADHD management, to assess risks of fibrosis, chronic inflammation, or incomplete breakdown. Fourth, artificial intelligence tools can be deployed to mine longitudinal data from electronic health records (EHRs). Machine learning and natural language processing models could accelerate the detection of rare or delayed safety signals among DDS-treated patients across hospital systems. Lastly, long-term patient-reported outcome (PRO) studies should be implemented using validated instruments and mobile platforms. For example, a decade-long follow-up through a mobile application could evaluate satisfaction, adherence, and functional outcomes in patients using smart transdermal patches for depression, providing insights into behavioral dynamics over time.

Collaborative and Policy Recommendations

Overcoming the long-term data challenge will require coordinated efforts from multiple stakeholders. Academic research consortia should take the lead in developing longitudinal study protocols and performing advanced statistical analyses. Regulatory bodies can play a key role by adopting adaptive approval models and strengthening post-market data requirements tailored to DDS. Pharmaceutical and device manufacturers must be willing to sponsor such efforts and participate in transparent data-sharing initiatives. Health technology assessment (HTA) agencies will also need to evaluate the cost-effectiveness of DDS based on long-term performance and help guide their inclusion in national formularies. In addition, policymakers can offer incentives to support extended evaluations, such as conditional reimbursement, fast-track review pathways, or extended market exclusivity for DDS that demonstrate durable safety and efficacy. These collaborative and policy-driven measures will be instrumental in building the robust evidence base necessary to support the long-term integration of DDS into mainstream neuropsychiatric care.

Interdisciplinary Collaboration in Drug Delivery System Innovation

The development and successful implementation of advanced drug delivery systems (DDSs) hinge on interdisciplinary collaboration. These technologies lie at the convergence of pharmaceutics, materials science, biomedical engineering, clinical pharmacology, and regulatory affairs, and require harmonized input from each of these domains. The complexity of DDS from design and manufacturing to regulatory evaluation and clinical use demands collaborative problem-solving that transcends traditional academic and industrial silos [94].

Challenges in Cross-Disciplinary Integration

Despite the clear necessity for integration, effective collaboration continues to be hindered by several factors. Differences in terminology, research culture, and development timelines across disciplines can create miscommunication and workflow misalignment. Moreover, incentives differ significantly: academia is primarily motivated by knowledge generation and publication, industry by product development and market returns, and clinicians by patient-centered outcomes. These divergent priorities complicate cooperative efforts. A further obstacle is the shortage of professionals with dual-disciplinary expertise, making it difficult to bridge technical and clinical domains effectively. Regulatory uncertainties, particularly in the evaluation of novel DDS, further discourage shared risk-taking and long-term cross-sector investment. Collectively, these barriers can delay innovation and impede the translation of promising DDS technologies into clinical practice.

Facilitating Cross-Sector Collaboration

Addressing these gaps requires strategic models that encourage early and active co-design among formulation scientists, biomedical engineers, clinicians, and regulators. Initiatives such as open innovation labs, translational research centers, and DDS-focused incubators can serve as collaborative environments to promote joint development. Real-world clinical feedback should be integrated into DDS development from the earliest stages through to post-marketing surveillance to ensure patient-centered refinement. Moreover, the cultivation of “hybrid professionals” trained in both technical and clinical disciplines is essential to facilitate effective communication and translational alignment. Examples of successful frameworks include NIH-funded translational hubs, academic-industry consortia, and partnerships like the European Innovative Medicines Initiative (IMI), which enable end-to-end therapeutic innovation with regulatory input built into the process [95].

Future Research and Innovation Proposals

To accelerate DDS innovation through interdisciplinary collaboration, several forward-looking strategies should be pursued. One promising initiative is the creation of integrated DDS co-innovation hubs, situated in academic medical centers where pharmaceutical scientists, engineers, clinicians, and regulatory experts co-develop new delivery platforms. These hubs, equipped with shared lab space and real-time feedback loops, would enable rapid design iteration and bridge the gap between research and clinical application. Another essential proposal is the establishment of cross-disciplinary fellowship programs - dual-domain PhD or postdoctoral tracks where candidates rotate through labs in pharmaceutical sciences, clinical departments, and engineering units. Such programs, ideally funded by NIH or industry foundations, would cultivate a future workforce fluent in the language and methods of multiple sectors. In parallel, structured regulatory-academic roundtables should be convened to jointly evaluate emerging DDS platforms. Annual forums co-hosted by agencies like the FDA or EMA and academic institutions would allow for early discussion of precompetitive data, surrogate endpoints, and combination product strategies, thereby reducing regulatory ambiguity and aligning expectations. Additionally, the development of open-access DDS simulation and testing libraries would democratize access to validated models, datasets, and simulation tools. For example, a global database with information on nano-biointeractions, skin permeation profiles, and depot release curves would lower barriers for small labs and startups to enter the DDS space. Finally, multisite collaborative demonstration projects should be launched to test real-world feasibility. A pilot study evaluating smart hydrogel-based DDS for chronic pain across diverse settings - academic hospitals, community clinics, and telehealth platforms - could offer robust insights into scalability, usability, and stakeholder buy-in.

Bridging Academia, Industry, and Policy

Achieving true interdisciplinary integration also demands a systemic bridge between academia, industry, and policy. Academic institutions must go beyond traditional research silos and commit to co-development throughout the product lifecycle, including regulatory engagement. Industry players should prioritize early-phase partnerships rather than focusing solely on late-stage licensing opportunities, thus supporting upstream innovation. Regulatory agencies have a key role in offering predictable and flexible evaluation pathways for novel DDS technologies and in promoting pre-competitive innovation through workshops and data-sharing initiatives. Clinicians, meanwhile, must be engaged not just during clinical trials but from the inception of DDS design, providing essential insights into patient behavior, usability challenges, and real-world clinical needs. Existing frameworks such as the Biomedical Advanced Research and Development Authority (BARDA) and the NIH’s National Center for Advancing Translational Sciences (NCATS) illustrate how multidisciplinary partnerships can successfully accelerate therapeutic innovation when well-structured and adequately resourced [96]. Figure 2 summarizes the challenges associated with the implementation of DDS in neuropsychiatric therapy.

## Conclusions

Advanced drug delivery systems (DDSs) have emerged as transformative tools in the treatment of neuropsychiatric disorders, offering innovative solutions to many of the limitations associated with conventional pharmacotherapy. By improving pharmacokinetic (PK) profiles, enhancing central nervous system (CNS) targeting, mitigating drug-drug interactions (DDIs), and promoting patient adherence, DDS enable more effective and safer psychotropic treatment regimens. Technologies such as nanoparticles, prodrugs, transdermal patches, depot injectables, and smart delivery systems have demonstrated their capacity to optimize therapeutic outcomes while minimizing systemic toxicity and dosing burden. Importantly, the integration of DDS with personalized medicine through pharmacogenomics, biomarker-responsive systems, and artificial intelligence marks a significant advancement toward individualized, data-driven neuropsychiatric care. These developments facilitate the tailoring of treatment to a patient’s unique genetic and metabolic profile, improving both clinical response and safety. Beyond their clinical utility, DDSs have the potential to reduce healthcare costs by minimizing hospitalizations, improving long-term outcomes, and enhancing medication adherence. However, challenges such as high development costs, limited accessibility in low-resource settings, complex regulatory pathways, and the need for long-term safety data must be addressed to fully realize their benefits. Future progress will depend on robust interdisciplinary collaboration across pharmaceutical sciences, bioengineering, clinical medicine, regulatory policy, and health economics. Global coordination through harmonized regulatory frameworks, collaborative research networks, public-private partnerships, and patient-centered education will be critical to ensuring equitable access to these advanced therapies. As innovation continues to accelerate, DDSs are poised to become a cornerstone of precision neuropsychiatric pharmacotherapy, offering customized, scalable, and sustainable solutions to some of the most challenging conditions in modern medicine.
